# Impaired Phasic Discharge of Locus Coeruleus Neurons Based on Persistent High Tonic Discharge—A New Hypothesis With Potential Implications for Neurodegenerative Diseases

**DOI:** 10.3389/fneur.2020.00371

**Published:** 2020-05-12

**Authors:** Kathrin Janitzky

**Affiliations:** Department of Neurology, Carl von Ossietzky University Oldenburg, Oldenburg, Germany

**Keywords:** locus coeruleus, phasic and tonic discharge, Alzheimers's disease, Parkinson's disease, norepinephrine, transcutaneous vagal stimulation, neurodegeneration, neuroprotection

## Abstract

The locus coeruleus (LC) is a small brainstem nucleus with widely distributed noradrenergic projections to the whole brain, and loss of LC neurons is a prominent feature of age-related neurodegenerative diseases, such as Alzheimer's disease (AD) and Parkinson's disease (PD). This article discusses the hypothesis that in early stages of neurodegenerative diseases, the discharge mode of LC neurons could be changed to a persistent high tonic discharge, which in turn might impair phasic discharge. Since phasic discharge of LC neurons is required for the release of high amounts of norepinephrine (NE) in the brain to promote anti-inflammatory and neuroprotective effects, persistent high tonic discharge of LC neurons could be a key factor in the progression of neurodegenerative diseases. Transcutaneous vagal stimulation (t-VNS), a non-invasive technique that potentially increases phasic discharge of LC neurons, could therefore provide a non-pharmacological treatment approach in specific disease stages. This article focuses on LC vulnerability in neurodegenerative diseases, discusses the hypothesis that a persistent high tonic discharge of LC neurons might affect neurodegenerative processes, and finally reflects on t-VNS as a potentially useful clinical tool in specific stages of AD and PD.

## Introduction

The locus coeruleus (LC) is a small nucleus located in the brainstem near the fourth ventricle and is composed of noradrenergic (NAergic) cells. Despite comprising only roughly 30,000–50,000 neurons in the adult human brain (1–4), the LC consists of extensively branched efferent axons that project throughout the brain and spinal cord (1, 5–14). LC neurons project to all layers of the cortex (15) and have dense projections to the hippocampus (16) as well as the frontal cortex (17).

As part of the ascending reticular activating system, the LC affects consciousness, wakefulness, and attentiveness (14) by projecting axons into the whole brain and thereby activating neural networks across many brain regions synchronously. There is evidence for a functional and topographic order within the LC. LC neurons in the dorso-rostral part project to the neocortex and the hippocampus, whereas more caudo-ventrally located neurons project to the cerebellum and the spinal cord (11, 18–21). Furthermore, tracing studies revealed that individual LC neurons receive input from 9 to 15 different brain regions indicating a largely integrative input to a single LC neuron. The release of NE locally from the soma of LC neurons activates somatodendritic α2-autoreceptors that inhibit neuronal activity via an auto-inhibitory mechanism (22, 23).

Since the loss of LC neurons is a shared feature of neurodegenerative diseases, especially Parkinson's disease (PD) and Alzheimers's disease (AD) (24–26), a better understanding of the role of the LC in AD and PD may provide important insights into the underlying mechanism of these neurodegenerative diseases.

The first part of this article focuses on the neuroprotective effects of NE. Next, it discusses potential mechanisms underlying the selective vulnerability of LC neurons to neurofibrillary tangles (NFTs)- and β-amyloid (Aβ) pathology in AD as well as α-synuclein (αSyn)-pathology in PD, respectively. Finally, a hypothesis discussing the potential relevance of changes in the discharge mode of LC neurons for neurodegeneration is presented together with t-VNS as a non-invasive technique to modulate LC activity with potentially neuroprotective effects.

## LC and Neuroprotection

Besides its role as a conventional neurotransmitter in the synapse, extrasynaptically released NE has a paracrine-type of anti-inflammatory and neuroprotective effect on surrounding neurons, glia cells and microvessels (1, 27, 28). Therefore, extrasynaptically released NE decreases toxin-induced inflammatory processes [for review, see (29, 30)], endotoxin-mediated inflammation (31) and Aβ induced neuroinflammation in the brain (24, 25, 30, 32–39). Furthermore, LC neurons innervate the cerebral vasculature throughout the brain via extensive varicosities for non-synaptic release of NE (38, 40), hence playing an important role in maintaining the blood-brain barrier (3, 38, 41, 42).

Moreover, LC neurons exhibit neuroprotective properties through the secretion of brain-derived neurotrophic factor (BDNF) and nerve growth factor (24, 32, 43). BDNF is synthesized in LC neurons (44, 45) and anterogradely transported and released from axon terminals in the projection areas in an activity-dependent manner (45–47). BDNF induces neurotrophic activity, promotes the survival of NAergic neurons and increases axonal sprouting of LC neurons at the terminal sites (45, 48, 49).

N-2-Chloroethyl-N-ethyl-2-bromobenzylamine hydrochloride (DSP-4), a selective neurotoxin for the LC-NAergic system in the rodent brain, is accumulated in NAergic nerve terminals, and damages them, thus resulting in rapid and long lasting loss of NE (50). DSP-4 increases the expression of proinflammatory factors, like inducible nitric oxide synthase (iNOS) (24, 30), and nitric oxide production, which in turn enhance the processing of amyloid precursor protein (APP) to Aβ (24, 51, 52). After treatment with the selective NAergic neurotoxin DSP-4, surviving LC neurons exhibit a regenerative axon sprouting in the target regions as a compensatory mechanism (45, 53), which seems to be important for the temporary maintenance of extracellular NE levels.

Thus, understanding the role of the LC in the process of neurodegeneration may start with the question: What makes LC neurons vulnerable to aging-related neuropathology?

## Potential Factors for the Vulnerability of LC Neurons

Why LC neurons are vulnerable to aging and aging-related neurodegenerative diseases, such as PD and AD, is not completely understood. One potential reason for the vulnerability of LC neurons might be intense mitochondrial demand caused by sustained cellular excitability attributable to autonomous pacemaking activity in these neurons, which results in mitochondrial dysfunction and cumulative oxidant stress (54–56).

A common feature of LC neurons is autonomous pacemaking. Small-conductance Ca^2+^-activated K^+^ (SK) channels are essential regulators of the intrinsic pacemaking of LC neurons and the activation of SK channels is primarily coupled to Ca^2+^ influx via the opening of L- and T-type calcium (Ca^2+^) channels (55, 57, 58). Activity-dependent Ca^2+^ entry through L-type Ca^2+^ channels enables feed-forward stimulation of mitochondrial oxidative phosphorylation, and thereby helps to prevent bioenergetic shortage when activity needs to be sustained, but in turn leads to basal mitochondrial oxidant stress (59). Hence, autonomous pacemaking caused by Ca^2+^ signaling in LC neurons requires elevated mitochondrial activity, leading to oxidative stress under basal conditions (55, 59–62), and thus resulting in elevated susceptibility to mitochondrial impairment.

Another potential factor for the vulnerability of LC neurons are their highly branched, long and thinly myelinated or unmyelinated axons that cause high energy demand, because ATP requirements for propagation of axon potentials grow exponentially with the level of branching (63, 64).

Oxidative stress together with required mitochondrial oxidative phosphorylation to sustain neurotransmitter release and cellular excitability, could interfere with key cellular functions, such as degradation of damaged and misfolded proteins (59), promoting protein aggregation and finally resulting in cell death. Subsequently, the brain is deprived of its NAergic innervation which may be a key step in the early stages of neurodegenerative diseases, such as AD and PD (64, 65).

## Physiology of LC Neurons

LC neurons show two different discharge modes, tonic and phasic. In the tonic mode, LC neurons show irregular but continuous firing patterns at 1–6 Hz whereas during the phasic mode, LC neurons fire in short (<300 ms) bursts of higher frequencies (10–15 Hz) that can occur spontaneously or associated with salient stimuli (1, 66, 67). Tonic discharge is high during stress and agitation, moderate during active wakefulness, low during drowsiness and completely absent during REM sleep (1, 66, 68, 69). Complete silencing of LC neurons during REM sleep may be due to elevated inhibitory GABAergic input from the ventral medulla (70–72). There is an inverted U-shaped correlation between tonic and phasic discharge, in such a way that phasic discharge to salient stimuli in the environment is optimal at a moderate tonic discharge level (1).

LC neurons are electrotonically coupled through gap junctions between dendrites outside of the nucleus, in the peri-coerulear region (73, 74). The strength of coupling changes between both discharges modes with increased coupling during phasic activation and decreased coupling in the tonic mode. The shift between the two discharge modes is thought to be modulated by the anterior cingulate (ACC) and the orbitofrontal cortices (OFC) of the prefrontal cortex (PFC) (1).

The PFC is important for a number of cognitive and executive functions (75), and strongly innervated and modulated by NAergic ascending projections from the LC. Aston-Jones and Cohen proposed that glutamatergic projections from the OFC and the ACC back to the LC are important in generating the patterns of LC activity (1). Besides, corticotrophin-releasing hormone (CRF)-containing afferences from the paraventricular nucleus of the hypothalamus and the central nucleus of the amygdala, increase tonic firing (74). Furthermore, LC neuronal activity is inhibited by local GABAergic interneurons, located dorsomedial to the LC nucleus, which hyperpolarize LC cells and reset their spontaneous activity (76).

## Stress-Induced Changes in LC Activity

Sustained tonic activity during waking is metabolically demanding and may render LC neurons a vulnerable target to stress (77). Particularly, stress-induced high tonic activity, mediated in part by the stress-related neuropeptide CRF, causes vulnerability to damage induced by high energy demands (33, 78) and makes LC neurons stress-sensitive. Stressful stimuli activate the hypothalamic-pituitary-adrenal axis and cause a release of CRF. CRF-immunoreactive fibers densely innervate the pericoerulear region that contains the dendrites of LC neurons (79) and CRF increases the tonic discharge of LC neurons (80–84). CRF peptide promotes the tonic discharge mode of LC neurons with a decreased maximum magnitude and slower onset, but a much longer duration of activity (74). Furthermore, stress seems to cause long-lasting changes in the LC that directly impact LC function and induce morphological alterations in LC neurons, such as proliferation of dendrites and axons (80, 85–94).

As a protection against these changes, stress-induced increase of NE release triggers auto-inhibitory mechanisms via α2-autoreceptors on LC neurons (78, 95, 96), which induce hyperpolarization and decrease the sensitivity of LC neurons to stimulation. This negative feedback mechanism protects LC neurons against stress-induced changes and damage of these autoregulatory mechanisms may contribute to neurodegenerative diseases like AD and PD (74, 78).

In conclusion, LC neurons are metabolically demanding and highly vulnerable to stress. Given that death of LC neurons is a shared feature of PD and AD (24–26), the damage of autoregulatory mechanisms that protect LC neurons from stress-induced changes might be involved in neurodegenerative processes of both diseases.

### LC and AD Pathology

Neurofibrillary tangles (NFTs) are aggregates of the microtubule-associated protein tau and increasing levels of tau pathology characterize the advancing stages in the development of AD. The LC is the first brainstem structure that displays pretangle material [for details see (97–100)], and thus axonal projections from the LC to the transentorhinal region could be important for the anterograde induction of tau pathology (97). Since aggregation of tau in the LC is one of the first pathological hallmarks of AD and precedes cortical tau pathology, it may act as a seed for subsequent spreading of tau pathology throughout the brain (38, 97, 98, 100–103). As hyperphosphorylated tau levels in the LC increase, the volume of the LC decreases in early stages of AD (102, 104). While total numbers of LC neurons are relatively stable until Braak stage II, they are significantly reduced in Braak stages III-VI, and analyses in the human brain revealed that as the Braak stage increased by one unit, the average LC volume decreased by 8.4% (38, 104). A loss of 30% characterizes the transition to MCI and a 55% reduction represents AD (105).

Since NE released from LC neurons is needed to maintain Aβ clearance, the progression of LC degeneration contributes to Aβ pathology in AD (36, 106), and further degeneration of LC neurons might be triggered by an Aβ-mediated failure in the anterograde and retrograde transport of neurotrophic factors like BDNF in LC axons (99, 107, 108). The bidirectional relationship between Aβ pathology and LC degeneration may lead to an exponential progression of the disease, because increased Aβ-levels exaggerate LC degeneration, which in turn reduces NE levels in the terminal fields that diminish the internalization of Aβ by microglia, adding to increased Aβ pathology (35). Progressive loss of LC neurons and the concomitant decrease of NE levels in the brain diminish anti-inflammatory and neuroprotective mechanisms and finally result in an exacerbation of Aβ-induced pro-inflammatory processes and neurotoxicity (31, 109), as well as tau pathology (101, 110).

NE deficiency in the cortex impairs the activation of microglia, the induction of their migration toward amyloid plaques and the stimulation of the internalization and clearance of Aβ (35). Furthermore, NE deficiency results in an increased tau phosphorylation and compromises neurogenesis in the dentate gyrus, dendritic arborization of new neurons and synaptic plasticity (111).

Smaller fusiform cells located in the dorsal part of the LC (23, 112, 113) that project to forebrain regions such as the PFC (23), are characterized by a high density of voltage-gated Ca^2+^ channels, which enables higher spontaneous firing frequencies (57). Furthermore, this subpopulation of PFC-projecting LC neurons is more excitable and responsive to glutamate than LC neurons projecting to other cortical circuitries (114). This subpopulation of LC neurons that can be distinguished from other LC neurons on the basis of their anatomical projections, molecular phenotypes, and electrophysiological properties, may be more vulnerable to activity-dependent cellular dysfunctions and the neurodegenerative processes in AD because of their higher basal discharge rates (114).

In summary, it can be concluded that NFT pathology causes dysfunction of LC neurons resulting in decreased NE levels in target regions, which contributes to Aβ pathology. This, in turn, accelerates LC degeneration and diminishes anti-inflammatory and neuroprotective effects of NE, resulting in increased Aβ plaque load. Therefore, LC degeneration and Aβ pathology synergistically interact to generate neurodegeneration in AD.

### LC and PD Pathology

In PD, α-synuclein (αSyn)-positive deposits, called Lewy bodies, can be found in the LC in Braak stage II, and thus earlier than in the substantia nigra pars compacta (SNc) (115, 116). It is believed that αSyn burden may lead to neuronal dysfunction and impaired neurotransmitter release, but αSyn pathology does not correlate well with cell death (2, 117, 118). Accordingly, αSyn may contribute to neurodegeneration in PD, but is not likely to be the sole reason (119). Furthermore, there is evidence that αSyn can be transferred across synapses and spread within postsynaptic cells in a prion-like fashion (3, 120).

A loss of LC neurons can be found throughout the rostral-caudal extent of the nucleus (20), earlier and in a greater magnitude as compared to the SNc (20, 121), with the onset of LC pathology occurring more than 10 years before the clinical diagnose of PD (122, 123).

Neuromelanin (NM) is an autophagic product synthesized via oxidation of catecholamines and subsequent reactions, and it is the main iron storage mechanism in neurons that protects them from iron-mediated neurotoxicity caused by superoxide free radicals (124–127). Although intraneuronal NM is neuroprotective, NM released by dying neurons can trigger neuroinflammation via activation of microglia (128). Postmortem studies have shown NM accumulation in LC neurons with increasing age (129). Several studies found an inverted U-shaped correlation between NM accumulation in LC cells and age with peak levels around 60 years, followed by an age-related decline related to loss of LC neurons (130, 131). However, some studies reported a linear age-related increase of NM in LC neurons without age-related decline (132). Nevertheless, under pathological conditions involving LC degeneration, the pigment is diminished (132, 133).

NM-sensitive Magnetic resonance imaging (NM-MRI) allows for *in vivo* visualization of the LC by exploiting the presence of NM (125, 134–138). Studies indicate that NM-MRI can detect structural alterations in the LC in early disease stages (139), even in patients with rapid eye movement (REM) sleep behavioral disorder (RBD) (140, 141), thus indicating NM-MRI as a potential biomarker in prodromal stages of neurodegenerative diseases [(138, 142) for a review see (143, 144)].

RBD is a prodrome of α-synucleinopathies, like PD, that appears 10 or more years before the first motor symptoms occur (145–147). Patients with RBD show fully developed αSyn pathology in the LC, equivalent to the pathology found in patients diagnosed with PD, while exhibiting normal nigrostriatal dopaminergic innervation (148). This begs the question if there are special characteristics of LC neurons, which are relevant to early αSyn pathology and Lewy body burden (106, 149–151).

One feature of LC neurons that may affect their sensitivity for PD-pathology is the presence of elevated Ca^2+^ concentrations in the cytosol (119). Autonomous pacemaking of LC neurons caused by voltage-sensitive Ca^2+^ channels (57) requires extensive Ca^2+^ entry to stimulate oxidative phosphorylation, which promotes high levels of reactive oxygen species (ROS) production, thus elevating oxidative stress (55, 152). Thus, sustained neurotransmitter release and neuronal excitability of LC neurons require high energetic demands that could impair other key cellular functions, such as degradation of misfolded proteins and promote their accumulation in intracellular inclusions (59). αSyn is widely expressed in the nervous system and located in presynaptic terminals, where it is involved in the regulation of synaptic vesicle exocytosis (153–157). Furthermore, αSyn interferes with Ca^2+^ homeostasis (59, 158–160), for instance by increasing ion permeability of the lysosomal and plasma membrane (161–163). Extracellularly accumulated αSyn increases the permeability of Ca^2+^ channels resulting in increased cytoplasmatic Ca^2+^ (119). Hence, αSyn oligomers are able to increase internal Ca^2+^ concentrations and Ca^2+^, in turn, increases αSyn oligomerization with cytotoxic effects (164–166). This positive feedback cycle of αSyn oligomerization and increased internal Ca^2+^ concentration could make LC neurons more vulnerable to PD pathology, because of Ca^2+^ channel-mediated pacemaking and the cellular burden associated with it (57). NE stabilizes αSyn in a soluble, monomeric form, thereby preventing the formation of toxic oligomers and enabling the disaggregation of existing fibrils (167). Consequently, decreased NE levels increase αSyn oligomerization, and therefore, LC degeneration and αSyn pathology synergistically interact to induce neurodegeneration in PD.

## Discussion

### Persistent High Tonic Discharge of LC Neurons and Potential Implications for Neurodegenerative Diseases

The findings mentioned above suggest that AD and PD are both characterized by a significant degeneration of LC neurons, despite having distinct pathologies (22, 106, 121). Postmortem studies reveal disease-specific patterns of LC cell loss, affecting the whole LC in PD. In AD, particularly the rostral and dorsal parts of the LC are affected, along with cortical-projecting LC neurons, while the caudal and ventral parts that contain non-cortical-projecting neurons are spared (20, 104). Slight differences in LC pathology in AD and PD could be a result of different underlying neuropathological mechanisms that may depend on the internal organization of the LC nucleus, the modulation of neuronal activity and the complexity of axonal projections of LC cells. The LC is not a single functional entity of neurons that function as a whole in order to control global arousal. Instead, LC neurons appear to be a collection of NAergic cells with sub-specializations according to their anatomical projections (114, 168–170), electrical properties (75, 168) and co-transmitter content, with distinctive roles in regulating brain function (168, 171).

The loss of LC neurons occurs earlier and in a greater magnitude than the atrophy of the hippocampus in AD and the loss of dopaminergic cells in the SNc in PD (25, 26, 104, 106, 121, 150, 172–175). However, LC pathology starts much earlier than evidence of cell loss. Grinberg and colleagues showed tau pathology in the LC in Braak stage 0, but no significantly decreased number of LC neurons until Braak stage III (104), indicating that LC neurons may survive substantial tau burden even with impaired NAergic neurotransmission (176). Moreover, αSyn pathology in the LC is evident early in the premotor phase of PD (Braak stage II), prior to the involvement of the dopaminergic SNc (Braak stage III) (177), but LC neurons can survive with αSyn pathology for years before significant cell loss is evident (20, 26, 121). Thus, tau- and αSyn pathology is verifiable in LC neurons many years before a significant loss of LC neurons is detectable in AD and PD, respectively (45, 106, 150, 174, 178–180).

Previous studies suggest that early damage to the LC in preclinical or prodromal AD may result in a persistent state of high tonic activity (181), which might be detrimental to brain functions that require phasic responses. Wang et al. (182) have shown that the mean firing rate of LC neurons increases significantly 2 and 4 weeks after unilateral lesion of the nigrostriatal pathway in the rat by local injection of 6-hydroxydopamine (6-OHDA) into the right substantia nigra pars compacta (SNc). Furthermore, the percentage of neurons with irregular firing patterns increased significantly. The authors postulated that 6-OHDA lesions of the SNc caused loss of LC neurons and decreased NE concentration in the LC of 6-OHDA-lesioned rats, which in turn resulted in overactivity of residual LC neurons. Moreover, patch-clamp data from Parkin-knockout mice also showed increased spontaneous firing of aged LC neurons caused by alterations in calcium-dependent excitability (183). In contrast, however, Miguelez et al. (184) found no effects of 6-OHDA infusion into the right medial forebrain bundle on the number of spontaneously active LC neurons, but in turn reported a significantly lower basal firing rate after 6-OHDA infusion and postulated that changes in firing rate may be attributed to dopaminergic degeneration. Another study showed an increase in α2-adrenoceptor mRNA in the LC in 6-OHDA-lesiond rats (185), and α2-adrenoceptors modulate the firing rate of the LC by inhibiting neuronal activity (186). With respect to these apparently opposing results, new mouse models, which overexpress αSyn in the LC seem to be a promising approach, since these models capture some cardinal morphological changes in human PD more closely (187, 188). Unfortunately, no electrophysiological LC data based on these models have been reported to date. The fundamental problem, however, off all the reported electrophysiological findings is that in all studies, recordings have been conducted under anesthesia, which impacts spontaneous LC activity (66, 189–192), and thus limits any conclusions about tonic LC discharge in awake animals, let alone patients.

Since recording from LC neurons is a challenging and invasive task, human data are lacking to date. Therefore, to make inferences about LC activity in humans, one has to rely on more indirect measures, like event-related potentials (ERPs), for instance the P300. The P300 is an event-related potential (ERP) that can be recorded in humans from the scalp in an auditory oddball paradigm. In contrast to former reviews that discuss glutamate as the most important neurotransmitter for the generation of the P300 as well as the cholinergic system and GABAergic influences as important modulators (193), more recent reviews suggest that dopamine and NE are the most important modulators for the generation of the P300 (194–196). The P300 can be devided into two subcomponents, the P3a and the P3b, respectively (196). P3a seems to depend on dopaminergic (DAergic) activity and is thought to reflect a novelty-driven orienting response to distractors that can be recorded more frontally on the scalp (194). On the other hand, P3b seems to be related to memory and decision making driven by phasic NAergic LC activity and can be recorded from more temporal-parietal areas (195, 196). Hence, a dual-transmitter P300 hypothesis was assumed that associated DAergic neurotransmission with P3a and LC-NAergic neurotransmission with P3b (194). Furthermore, Nieuwenhuis et al. (195) suggest that the P300 reflects phasic activity of the LC-NAergic system, and recent studies suggest that phasic LC activity depends on background tonic discharge of LC neurons in an inverted U-shaped manner, with highest phasic discharge rates at moderate levels of tonic LC activity, which would in turn create the largest P3b amplitudes (1, 197).

Also, animal studies suggest that the P300 can be interpreted as a cortical correlate of the phasic LC response (195, 197–199), and the hypothesis that phasic LC activity contributes to P300 generation during a target detection task is consistent with the fact that both phasic LC activity and the P300 depend on the motivational significance of the eliciting stimulus as well as underlying attentional mechanisms and show congruent latencies in response to target stimuli [for review see (195)]. Hence, it was suggested that the P300 reflects NE mediated enhancement of gain in the cerebral cortex induced by phasic LC activity and thus enhances cortical encoding of salient stimuli (1, 67, 195, 198).

In addition, studies in PD patients have shown a reduced P300 amplitude (200–206) or an increase in P300 latency (207, 208), whereas in AD patients an increase in P300 latency was found (209–213). If the hypothesis given above is correct and an optimal rate of phasic LC activity is contingent upon a moderate level of tonic activity (1), then a reduction in P300 amplitude and/or increase in P300 latency might reflect impaired phasic activity caused by persistent high tonic LC activity.

Furthermore, persistent high tonic discharge of LC neurons during REM sleep could explain RBD, an early feature of neurodegenerative disorders, including PD (214). RBD is characterized by REM sleep without atonia (RSWA), leading an individual to “act out” dreams, and lesions of the LC and subcoeruleus (SubC) nucleus complex have been theorized to be one possible cause of RBD (215, 216). Glutamatergic neurons in the ventral part of the SubC project to the ventromedial medulla and the spinal cord, who's GABA and glycine neurons inhibit motoneurons and initiate REM sleep atonia (214). These glutamatergic neurons in the SubC get inhibitory afferents from NAergic LC neurons (217, 218). Because normally, tonic discharge of LC neurons is completely absent during REM sleep (66), the SubC is not inhibited by the LC during that state and can thus promote REM sleep muscle paralysis. Therefore, a constantly high tonic discharge of LC neurons during REM sleep could result in an over-inhibition of the SubC and thus explain RSWA.

Moreover, persistent high tonic LC activity during sleep could contribute to the accumulation of protein aggregation and promote neurodegeneration by an inhibition of the glymphatic system (219). The glymphatic system is a macroscopic waste clearance system formed by astroglial cells (220). It is turned on during sleep and enables the elimination of potentially neurotoxic waste products, including Aβ [for details see (219)]. Hence, dysfunction of the glymphatic system could thus contribute to the accumulation of misfolded or hyperphosphorylated proteins and could thereby render the brain more vulnerable to developing a neurodegenerative pathology, because all neurodegenerative diseases are characterized by accumulation of aggregated proteins (221), e.g., misfolded Aβ and NFT in AD or misfolded αSyn in PD, respectively.

A distinct subpopulation of LC neurons in the dorsal part of the nucleus that innervates the PFC (168, 222) and the hippocampus (170), seems to be particularly vulnerable to the pathophysiological processes in AD. Since these LC cells are characterized by greater synaptic excitability, higher spontaneous firing frequencies, and higher susceptibility to glutamate (75), they could be more vulnerable to stress and hyperactivity-dependent cell death. If LC neurons in the dorsal part of the nucleus are indeed the first set of neurons affected by the pathophysiological processes, then this might result in persistent high tonic activity and impaired task-related phasic activity in those cells (181), which in turn may result in compromised PFC and hippocampal functions (64, 176).

Transient silence of LC neurons during REM sleep and prior to each non-REM sleep spindle seems to be important for synaptic plasticity and essential for hippocampus-dependent memory consolidation (223). Swift and colleagues could show that increased LC activity during sleep has no effect on the stability and duration of sleep states, but impairs learning related signatures of non-REM and REM sleep (223). Since glutamatergic projections from the SubC to the medial septum innervate the hippocampus and promote the generation of the theta electroencephalographic activity characterizing REM sleep (218), a persistent high tonic discharge of LC neurons could impair theta oscillation in the hippocampus during REM sleep, which has been shown to be important for consolidation of hippocampal-dependent memories (224, 225).

Since LC neurons in the dorsal part of the nucleus show minimally overlapping projections to the OFC, the medial PFC, and anterior cingulate (ACC) cortex (75), respectively, neuronal activity in individual prefrontal subregions could also be impaired, resulting in compromised cognitive and executive functions, e.g. shifting of the attentional focus and behavioral adaptation in a changing environment. NAergic projections of LC neurons to the PFC are critical for the ability to rapidly switch attention between stimuli and stimulus categories (226–229), and therefore tests of cognitive flexibility could possibly be used to determine LC dysfunction in early stages of AD (230) and PD (231).

### Consequences of Persistent and Abnormally High Tonic Discharge of LC Neurons

Neuronal plasticity is essential to adapt to a changing environment through strengthening, weakening or adding of synaptic connections or by promoting neurogenesis (232). Alteration in synaptic plasticity is an early feature in AD with abnormally suppressed efficacy of neuronal plasticity linked to cognitive decline (232). Since deprivation of cortical NAergic innervation is associated with reduced expression of genes important for synaptic plasticity in the cerebral cortex, the NAergic system seems to have a gating function for neuronal plasticity (233). Oberman and Pascual-Leone (232) hypothesize that cortical “hyperplasticity” in autism spectrum disorder (ASD) may provide protection for this population against the development of age-related cognitive decline and AD, and it was assumed that in autism, tonic LC discharge is affected, which in turn has a protective effect against later development of AD (234).

Assuming a persistent and abnormally high tonic discharge of LC neurons in the dorsal part of the nucleus in early stages of AD (20, 64, 104, 170, 181), phasic discharge of these cells—which requires a moderate tonic discharge level—could be impaired as a result. Because the release of high amounts of NE requires phasic spiking of LC neurons (32, 65, 235–238), NE levels in the cortex might not be high enough in this scenario to activate low-affinity β-adrenoceptors that facilitate the anti-inflammatory and neuroprotective effects of NE (32, 35, 65). It can be assumed that sustained high tonic activity would induce compensatory mechanisms that could initially help to maintain homeostasis (85, 89, 92–94), such as axon sprouting in the terminal fields, augmented synthesis of NE by increased activity of tyrosine hydroxylase and dopamine beta-hydroxylase, and decreased NE transporter activity (174, 180, 239–242). All these compensatory mechanisms, however, would further serve to increase the energy demand and oxidative stress of LC neurons. As a protection against these changes, NE could trigger auto-inhibitory mechanisms via α2-autoreceptors on LC neurons (78, 95, 96), which induce hyperpolarization and decrease the sensitivity of LC neurons to dendritic stimulation. These autoregulatory mechanisms may protect LC neurons, but could on the other hand interfere with LC functions, because they further impair the ability of LC neurons to create selective phasic responses, as described above. Subsequently, sustained increase of basal mitochondrial oxidant stress in tonically discharging LC neurons could contribute to an impaired capability to maintain other key cellular functions, such as the degradation of damaged or misfolded proteins (59), giving rise to axon terminal degeneration as an adaptation to excessively high metabolic demand. The consequences of decreased NE levels in the PFC and hippocampus are increased neuro-inflammation and neurodegeneration, and finally, increased amounts of damaged axonal proteins, such as αSyn and tau-protein may promote aggregation and accelerate cell death of LC neurons. If a dysbalance between tonic and phasic discharge of LC neurons is part of the problem—due to the fact that sustained high tonic discharge impairs phasic discharge to salient stimuli—then modulation of afferent stimulation may be an option to normalize LC functions.

### t-VNS, a Potential Non-invasive Technique to Increase Phasic Discharge of LC Neurons

Transcutaneous vagal stimulation (t-VNS) is a novel non-invasive brain stimulation technique that increases activation of the LC and NE release (243), via activation of the auricular branch of the vagal nerve at the external ear (244), and fMRI studies in humans show that t-VNS and invasive vagal nerve stimulation (i-VNS) activate the same afferent vagal projection sites (245). The vagal nerve innervates the nucleus of the solitary tract (NTS), which directly modulates the activity of LC neurons via monosynaptic excitatory projections (246, 247) and indirectly excites the LC via the nucleus paragigantocellularis, providing pathways by which VNS could directly drive short latency spiking in the LC (248, 249). Stronger activation of LC dendrites in the peri-coerulear area via t-VNS may significantly influence the activity of LC neurons, for instance via changes in their electrotonic coupling (250) due to gap junctions between dendrites in the peri-coerulear region (73, 74). Previous studies show that when LC neurons are isolated from the peri-coerulear dendritic region, synchronous activity is reduced or abolished (73). This suggests that the modulation of electrical interactions between dendrites in the peri-coerulear area can stimulate synchronous activity within the LC. Hence, t-VNS may increase electrotonic coupling, and in that way may promote phasic activation and decrease tonic activation of LC neurons, which in turn could normalize the dysbalance in LC activity and facilitate phasic spiking of LC neurons required for the release of NE levels high enough to activate low-affinity β-adrenoceptors to promote anti-inflammatory and neuroprotective mechanisms (32, 35, 65).

Electrophysiological studies in rats show that i-VNS increases the firing rate of LC neurons above their baseline activity (246, 251–253). Moreover, VNS causes a significant increase in the percentage of LC neurons firing in bursts (251–253), which in turn leads to a greater NE release in terminal fields as compared to single pulses (254). Short trains of VNS drive rapid, phasic neural activity in the LC (248), and 30 second trains of VNS increase firing rates of LC neurons and NE concentrations in the cortex and hippocampus on the order of minutes to hours (246, 248, 251, 253, 255).

Since direct measurements would require invasive procedures there is a lack of human data on LC activity. However, a recent review of Burger and colleagues discusses the P300 as a potential biomarker for t-VNS effects (256) that seems to represent the phasic activity of the LC-NAergic system. Studies investigating t-VNS effects on P3b in an oddball paradigm in healthy subjects found increased P300/P3b amplitudes compared to sham stimulation (257, 258). Furthermore, consistent with the hypothesis that P3b reflects the activation of the LC-NA system, no effects of t-VNS stimulation on P3a were found (257, 259). However, there are other studies that could not confirm those data (260, 261).

**Figure 1:**
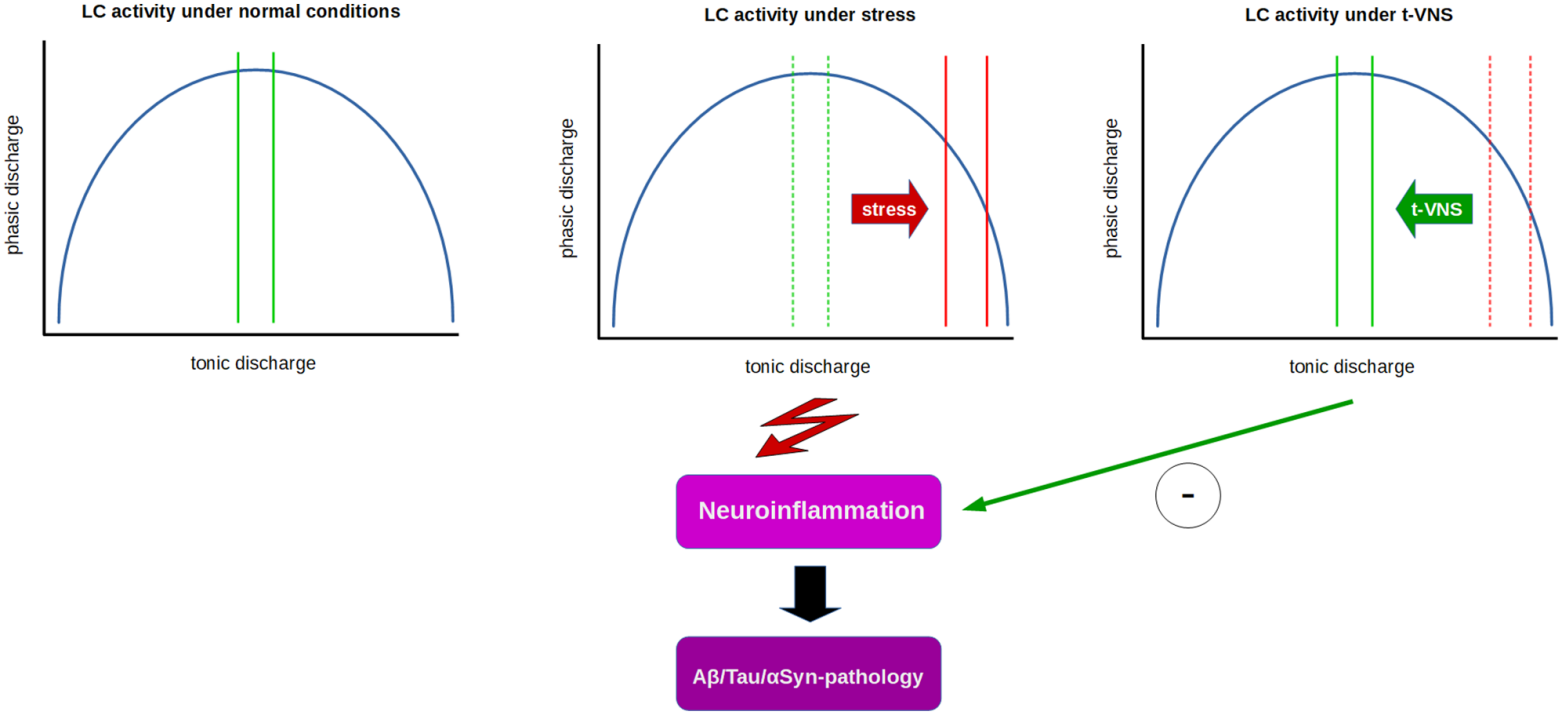
Relation between tonic and phasic LC discharge (blue curve) and proposed effects of t-VNS treatment. Under normal conditions **(left)**, a moderate range of tonic activity (indicated by green lines) allows for an optimal level of phasic activity in response to salient stimuli. Under constant stress **(middle)**, LC tonic activity is thought to shift from moderate to high tonic discharge (solid red lines) which in turn impairs phasic discharge, presumably facilitating neuroinflammatory processes and contributing to neurodegeneration. t-VNS treatment **(right)** is proposed to restore tonic discharge to a moderate range, thus enabling an optimal level of phasic discharge, and presumably inhibiting neuroinflammatory/neurodegenerative processes by neuroprotective effects of NE.

If phasic LC activity in humans could be assessed by P3b measurements, a reduction in P3b amplitude and/or increase in latency in PD or AD patients could possibly indicate t-VNS as a valid non-invasive treatment option. Indeed, we have shown that t-VNS in healthy subjects leads to an increase in P3b amplitude and a reduction in P3b latency (258). Based on the literature cited above, these findings might be interpreted as t-VNS having a positive influence on the imbalance between tonic and phasic discharge by shifting LC activity toward increased phasic activation. If this presumption can stand the test of further experimental scrutiny, then t-VNS may have potential as a clinical tool used to normalize the imbalance between tonic and phasic LC activity in patients in certain stages of neurodegenerative diseases, and possibly have additional benefits by promoting anti-inflammatory and neuroprotective effects which require sufficiently large NE levels related to phasic LC discharge (32, 35, 65, 262). Recently, auricular t-VNS has been shown to have neuroprotective effects on dopaminergic neurons in 6-OHDA-treated rats and the authors suggested that these effects might be related to the inhibition of neuroinflammation (262). Thus, these results indicate t-VNS as a prospectively useful tool with potential anti-inflammatory and neuroprotective effects in early stages of neurodegenerative diseases, like AD and PD (Figure 1).

### Concluding Remarks

Studies suggest that early damage to the LC in preclinical or prodromal phases of neurodegenerative diseases, such as AD and PD, may result in an abnormally persistent state of high tonic activity of the LC (181) that impairs phasic discharge, which requires a moderate tonic activity level. Since phasic LC discharge is essential for optimization of cognitive and neural network function (67), as well as anti-inflammatory and neuroprotective effects, a potential facilitation of phasic LC activity by t-VNS might be a useful clinical tool in early stages of neurodegenerative diseases, like AD and PD (Figure 1).

## Data Availability Statement

The raw data supporting the conclusions of this article will be made available by the authors, without undue reservation, to any qualified researcher.

## Author Contributions

KJ developed the presented hypotheses and wrote the manuscript.

## Conflict of Interest

The author declares that the research was conducted in the absence of any commercial or financial relationships that could be construed as a potential conflict of interest.

## Abbreviations

AD: Alzheimers's disease
Aβ: amyloid-beta
ACC: anterior cingulate cortex
αSyn: α-synuclein
BDNF: brain derived neurotrophic factor
CRF: corticotrophin-releasing hormone
LC: locus coeruleus
NA: noradrenaline
NE: norepinephrine
NFT: neurofibrillary tangles
NTS: nucleus of the solitary tract
OFC: orbitofrontal cortex
PD: Parkinson's disease
PFC: prefrontal cortex
RBD: rapid eye movement sleep behavioral disorder
SNc: substantia nigra pars compacta
SubC: subcoeruleus
t-VNS: transcutaneous vagal stimulation.
